# Epidemic Poliomyelitis—Joint Session of American Orthopedic and American Pediatric Societies Take Action—Health Boards Urged to Study Epidemiology of the Disease

**Published:** 1910-09

**Authors:** Robert W. Lovett, Irving M. Snow

**Affiliations:** President; Committee on Poliomyelitis, American Orthopedic and Pediatric Societies; Secretary; No. 475 Franklin St., Buffalo, N. Y.


### CORRESPONDENCE

Epidemic Poliomyelitis—Joint Session of American Orthopedic and American Pediatric Societies take action—Health Boards urged to study Epidemiology of the Disease.

*Editor Buffalo Medical Journal*:

Sir—At the recent meeting of the Congress of American Physicians and Surgeons held at Washington, in May, 1910, a joint session of the American Orthopedic and American Pediatric Societies was held and the subject of epidemic poliomyelitis was discussed. The following resolution was adopted:

It having been shown by recent epidemics and investigations connected with the same that epidemic infantile spinal paralysis is an infectious communicable disease that has a mortality of from 5 to 20 per cent., and that 75 per cent. or more of the patients surviving are permanently crippled, state boards of health and other health authorities are urged to adopt the same or similar measures as are already adopted and enforced in Massachusetts, for ascertaining the modes of origin and manner of distribution of the disease with a view of controlling and limiting the spread of so serious an affection.

A committee with Dr. Robert W. Lovett, president, Boston, Mass., and Dr. Irving M. Snow, secretary, Buffalo, N. Y., was appointed to urge the various state and municipal health authorities to take up the work of investigation of the various foci of epidemic poliomyelitis, to study its epidemiology and to instruct the public that the disease is at least mildly communicable.

May we ask you to publish this letter and the resolutions in your journal and also to allude to the matter editorially, urging the Health Commissioners of the various states of the United States and of the provinces of Canada to follow the example of the Massachusetts Health Department in studying the epidemiology of poliomyelitis.

August 11, 1910.

Robert W. Lovett, M.D., President,
Committee on Poliomyelitis, American Orthopedic and Pediatric Societies.

Irving M. Snow, M.D., Secretary,
No. 475 Franklin St., Buffalo, N. Y.

---

"You're in for a siege," declared the fashionable physician.

"Quit your kidding," responded the malefactor of great wealth.

"It's a fact. You're going to be sick. You have violated nature's laws, and must pay the penalty."

"Aw, can't you get me off on a technicality, doc?"—*Louisville Courier-Journal.*

